# Evaluation of serological response to anti-SARS-CoV-2 mRNA vaccination in hematological patients

**DOI:** 10.3389/fimmu.2022.892331

**Published:** 2022-08-08

**Authors:** Sara Pasquina Pascale, Roberta Nuccorini, Teresa Pierri, Roberta Di Mare, Lucia Fabio, Emilia Lerose, Maria Antonietta Merlino, Pietro Schiavo, Angela Amendola, Gino Brucoli, Maria Denise Caputo, Ida Chitarrelli, Michele Cimminiello, Sabrina Coluzzi, Nunzio Biagio Filardi, Angela Matturro, Domenico Vertone, Monica Poggiaspalla, Francesco Malaspina, Gerardo Musuraca, Gennaro Coralluzzo, Clara Mannarella, Clelia Musto, Angela Pia Bellettieri, Giovanni Martinelli, Claudio Cerchione, Michele Pizzuti

**Affiliations:** ^1^ UOC di Ematologia, Azienda Ospedaliera Regionale “San Carlo”, Potenza, Italy; ^2^ UO di Medicina Trasfusionale, Azienda Ospedaliera Regionale “San Carlo”, Potenza, Italy; ^3^ Hematology Unit, IRCCS Istituto Romagnolo per lo Studio dei Tumori (IRST) “Dino Amadori”, Meldola, Italy; ^4^ UOS di Ematologia, Presidio Ospedaliero “Madonna delle Grazie”, Matera, Italy; ^5^ Direzione Sanitaria, Azienda Ospedaliera Regionale “San Carlo”, Potenza, Italy

**Keywords:** SARS-COV-2, mRNA vaccination, anti-spike IgG, hematological malignancies, onco-hematology

## Abstract

**Introduction:**

In immunocompromised patients, SARS-CoV-2 mRNA vaccine has been used in Italy from the beginning of the vaccination campaign, but several studies have shown that the serological response of onco-hematological patients was reduced compared to healthy subjects, due to the state of immunosuppression because of both underlying disease and administered therapy.

**Methods:**

We evaluated the association of anti-SARS-CoV-2 spike IgG titers in 215 hematological patients with clinical and demographic variables to verify if it was possible to identify predictive parameters of serological response, as well as using a control group, consisting of healthy health workers of San Carlo Hospital in Potenza. Anti-SARS-CoV2 IgG titers were evaluated after 30–45 days post second dose vaccine using chemiluminescent microparticle immunoassay technology.

**Results:**

Patients with hematological malignancies, compared with the control arm, had both a mean concentration of anti-SARS-CoV-2 IgG significantly lower and a seroconversion rate numerically lower. All chronic lymphatic leukemia patients showed levels of antibody titer below the mean concentration, also in only clinical surveillance patients. Comparing serological response in hematological malignancies, only acute leukemia patients who were off therapy had the highest seroconversion rate among the patients’ cohorts and a mean antibody concentration greater than the control arm. Patients treated with steroids and rituximab showed a lower level of anti-SARS-CoV-2 spike IgG. Differences in anti-spike IgG levels among chronic myeloid leukemia patients stratified according to tyrosine kinase inhibitor therapy and molecular response were observed, and they could have interesting implications on the evaluation of the effects of these drugs on the immune system, but having not reached statistical significance at the moment. The cohort of patients who received a stem cell transplant was very heterogeneous because it included different hematological malignancies and different types of transplant; however, a mean concentration of anti-SARS-CoV2 IgG greater than the control arm was reported. Indeed, among patients who performed a transplant for over 6 months only one had a spike IgG concentration below the cutoff.

**Conclusions:**

Our data confirm reduced serological response in hematological patients after anti-SARS-CoV-2 vaccination. However, we found a great diversity of SARS-CoV-2 antibody response according to types of pathologies and therapies.

## 1 Introduction

The novel coronavirus disease 2019 (SARS-CoV-2) pandemic emerged in Wuhan City, Hubei Province, China, on December 2019 which has spread throughout the world with over 4.5 million deaths globally and more than 130,000 in Italy.

Broad variations in practice treatment strategies have emerged during these months; patients had been treated, because of lack of effective antiviral agents, with therapies aimed above all at counteracting the complications induced by the infection such as corticosteroids, low-molecular-weight heparin, oxygen therapy, interleukin-6 inhibitors, and antibiotics, but the results have often been disappointing (1–3).

The use of SARS-CoV-2 convalescent plasma has provided contrasting results (4). Anti-SARS-CoV-2 monoclonal antibodies that target different proteins of SARS-CoV-2 have been shown to have a clinical benefit in treating SARS-CoV-2 infection especially if administered on the first days of infection, but their use is still limited (5, 6).

The only weapon to combat, on a large scale, the spread of SARS-CoV-2 has been vaccination. This has been performed with fundamentally two different vaccine technologies used in parallel: one based on SARS-CoV-2 mRNA (7, 8) and the other based on adenoviral vector (9, 10), both of which are capable of inducing the production of native viral spike proteins of SARS-CoV-2 and subsequently neutralizing antibodies.

Initially, also in Italy, both types of vaccines have been used; subsequently, vaccines based on adenovirus, due to the occurrence of severe atypical thrombotic phenomena, even if limited to a few cases, have been reserved for men and women older than 60 years and then gradually abandoned.

Real-life results have confirmed those obtained by registration studies with demonstration of good immunization obtained in vaccinated subjects.

In immunocompromised patients, the mRNA vaccine has been used in Italy from the beginning of the vaccination campaign, but several studies have shown that the serological response of onco-hematological patients was reduced compared to healthy subjects, due to the state of immunosuppression because of both underlying disease and administered therapy (11, 12).

In our study, we evaluated the association of anti-SARS-CoV-2 spike IgG titers in hematological patients with clinical and demographic variables to verify if it was possible to identify predictive parameters of serological response, as well as using a control group, consisting of healthy health workers of San Carlo Hospital in Potenza, to identify any differences in the response to the vaccine.

## 2 Patients and methods

A total of 215 patients, 128 men and 97 women, aged between 19 and 92 years, affected by different hematological malignancies and autoimmune disorders as listed in **Table 1**, treated at UOC of Hematology of San Carlo Hospital in Potenza, were included in the study. Patients who had previous exposure to the natural SARS-CoV-2 virus documented by a qualitative analysis of SARS-CoV-2-RNA on nasopharyngeal swab were excluded by the study. All of them had received the two FDA-recommended doses of the mRNA vaccine (BNT162b2) (7).

**Table 1:** Clinical characteristics of patients.

No.	215
Age, median (range)	65 (19-92)
Sex	
Male (%)	122 (57)
Female (%)	93 (43)
Type of hematological malignancies	
Acute leukemia (%)	17 (8)
Chronic myeloid leukemia (%)	44 (20)
Chronic lymphocytic leukemia (%)	17 (8)
Hodgkin’s lymphoma/non-Hodgkin’s lymphoma (%)	43 (20)
Plasma cell disorders (%)	47 (22)
Myeloproliferative neoplasm (%)	20 (9)
Myelodysplastic syndrome (%)	18 (8)
Autoimmune disorder (%)	9 (4)
Type of treatment	
Anti-CD-20 antibody	30
Tyrosine kinase inhibitor	44
Corticosteroids	70
Type of hematopoietic stem cell transplant	
Autologous	20
Allogeneic	10
SARS-CoV-2 vaccine	
BNT162b2	215 (100%)
Days between second vaccine dose and final outcome measurement, median (range)	36 (30-45)

The characteristics of patients are listed in **Table 1**. Since at UOC of Hematology, onco-hematologic diseases were mainly treated, the group of autoimmune disorders was poorly represented. We considered for each patient the phase of disease at the time of their vaccination. Eighty-six patients (40%) had an active malignancy and therapy underway, 23 patients (11%) had an active disease diagnosis, and 106 patients (49%) were in remission of disease. All patients affected by acute leukemia were in remission of disease, and they were off therapy (i.e., clinical surveillance) while among transplanted patients (20 autologous and 10 allogeneic) all but one underwent vaccination after 6 months from transplantation of hematopoietic stem cells.

We investigated the association between the quantitative concentration of anti-SARS-CoV-2 spike IgG and different hematological malignancies, type of treatment (focusing on corticosteroids, anti-CD20, and tyrosine kinase inhibitors (TKIs) for chronic myeloid leukemia (CML) patients and transplantation of hematopoietic stem cells), local and systemic side effects induced by vaccine, and the amount of immunoglobulins A, G, and M (IgA, IgG, IgM) in the blood.

A comparison with a sample of healthy health workers of San Carlo Hospital in Potenza was foreseen. This latter population was composed of 942 men and 1,680 women aged between 23 and 77 years, and they had completed the full vaccination cycle in January and February 2021. They, according to the internal policy of surveillance, performed periodical nasopharyngeal swab to investigate the presence of SARS-CoV-2 virus, and as for patients also healthcare workers who had a previous exposure were excluded by the analysis of the study. They had the function of representing the control arm, given they had received the same type of mRNA vaccine (BNT162b2), and anti-SARS-CoV-2 IgG titers were evaluated by the same method and after the same timing.

### 2.1 Serological test

Anti-SARS-CoV-2 IgG titers were evaluated 30–45 days after the second dose of vaccine using the AdviseDx SARS-CoV-2 IgG II assay (Abbott). This assay is an automated, two-step immunoassay for the qualitative and semiquantitative detection of IgG antibodies to SARS-CoV-2 in human serum and plasma using chemiluminescent microparticle immunoassay (CMIA) technology. The cutoff is 50.0 AU/ml.

### 2.2 Statistical analysis

Preliminarily, a descriptive univariate statistical analysis was carried out, consisting of percentages, mean, median, standard deviation, range, and interquartile difference calculation. The main results obtained were represented by appropriate graphs and diagrams.

Subsequently, an inferential statistical analysis was performed using, where appropriate, parametric and non-parametric statistical tests. In particular, for the comparison between means belonging to different groups, the Student’s t test was applied or, for more than two groups, the one- or two-way analysis of variance. The association between qualitative or nominal variables was evaluated by Pearson’s chi-square test or, in case of limited cases, by Fisher’s exact test. To evaluate the linear dependence between quantitative variables, the Bravais Pearson correlation coefficient and subsequent definition of linear regression models were used. As usual, the level of first alpha error was 0.05.

Finally, a multivariate approach was used with the definition of multiple logistic regression models. The variables found to be significant in inferential analysis were inserted into each model through two distinct selection methods: enter (altogether) or stepwise (from the strongest to the weakest). For each model, the Wald test, the level of significance and the odd ratio were calculated with the 95% confidence interval.

The collected data were archived on a Microsoft Excel spreadsheet. For statistical processing, the SPSS for Windows program (IBM, Release 25, 2019) was used.

### 2.3 Ethics statement

All procedures performed in this study were in accordance with the ethical standards of the Regional Ethics Committee for Basilicata (approval n° 42/2021), with the 1964 Helsinki declaration and its later amendments and with Good Clinical Practice (GCP) guidelines. Written informed consent was obtained from all individual participants included in the study.

## 3 Results

### 3.1 Comparison of serological response between hematological patients and the control group

In the control group, the mean concentration of anti-SARS-CoV-2 IgG was 11,842.8 AU/ml (range 0–40,000 AU/ml), with levels of antibodies significantly greater in women than in men (p < 0.0001) (**Table 2**) in accordance with literature data. Indeed, gender differences have been detected in both innate and adaptive immune responses, where women exhibit a higher humoral and cell-mediated immune responses than men (13, 14).

**Table 2:** Characteristics of the control group.

No.	2,622
Age, median (range)	50 (23-77)
Sex	
Male (%)	942 (36)
Female (%)	1,680 (64)
SARS-CoV-2 vaccine	
BNT162b2	2,622 (100%)
Days between second-dose vaccine and final outcome measurement (range)	38 (30-45)
IgG anti-SARS-CoV2, mean (range) (AU/mL)	11,842.8 (0-40,000)
Male IgG anti-SARS-CoV2, mean (range) (AU/mL)	10,738 (0.9-40,000)
Female IgG anti-SARS-CoV2, mean (range) (AU/mL)	12,477 (0-40,000)

**Figure 1:**
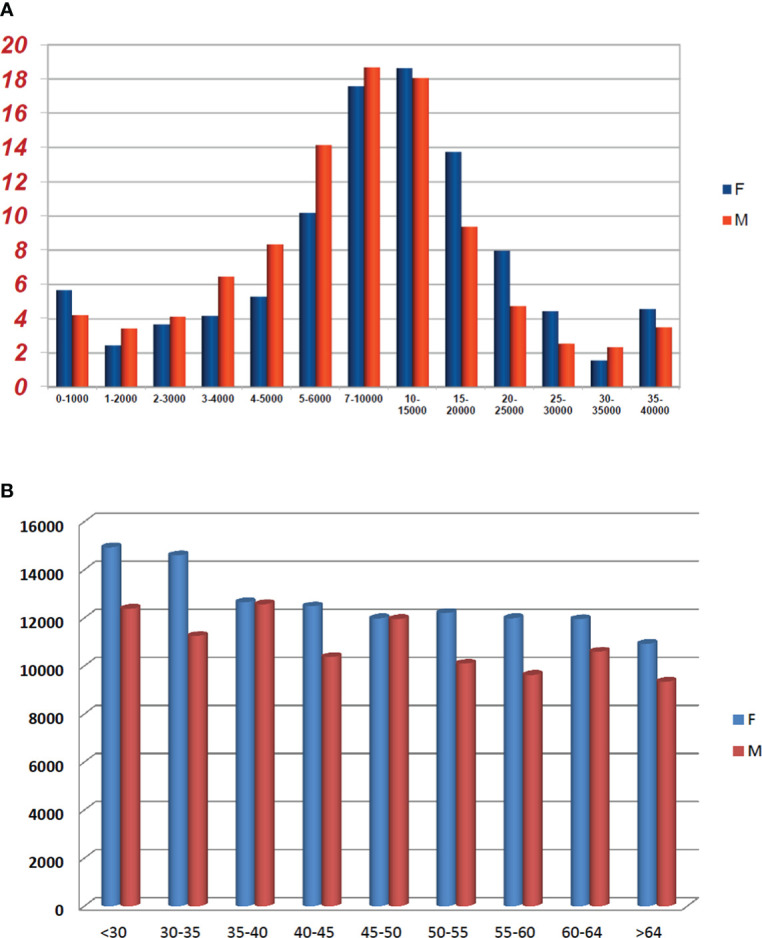
Comparison between sexes in the control group according to anti-SARS-CoV-2 concentrations **(A)** and age **(B)**. **(A)** Proportion of healthy subjects by sex and different IgG anti-SARS-CoV-2 concentration (AU/mL × 10^3^) groups. **(B)** IgG anti-SARS-CoV-2 mean concentration stratified by sex and age groups.

In **Figure 1**, differences in anti-SARS-CoV-2 IgG concentrations according to sex were shown; men were more numerous in the range 1,000–10,000 AU/ml while women were more in the range 10–40,000 AU/ml, and this difference between men and women was confirmed also within different age groups.

Patients had significantly lower antibody concentrations compared with the control arm (mean concentration 6,779.3) (p < 0.0001); also considering only patients younger than 70 years, the mean concentration of anti-SARS-CoV-2 IgG was lower than in the control arm (8,298 *vs*. 11,842.8 AU/ml).

The serological response was defined by the method at a cutoff of 50.0 AU/ml. In the control group, only 1.1% were not responders while in the study cohort the proportion of non-responders were significantly higher than in the control arm (17.6% *vs*. 1.1%) (p < 0.0001).

### 3.2 Comparison of serological response among different phases of disease

Patients in remission of disease had a significantly higher level of anti-SARS-CoV-2 IgG than patients with active malignancy and therapy underway (p = 0.045, **Supplemental Figure 1**). This lower seroconversion rate of patients with active disease could be due to both immune suppression related to disease and immune suppressive effects of disease-directed therapies.

### 3.3 Comparison of serological response among different hematological malignancies

**Figure 2:**
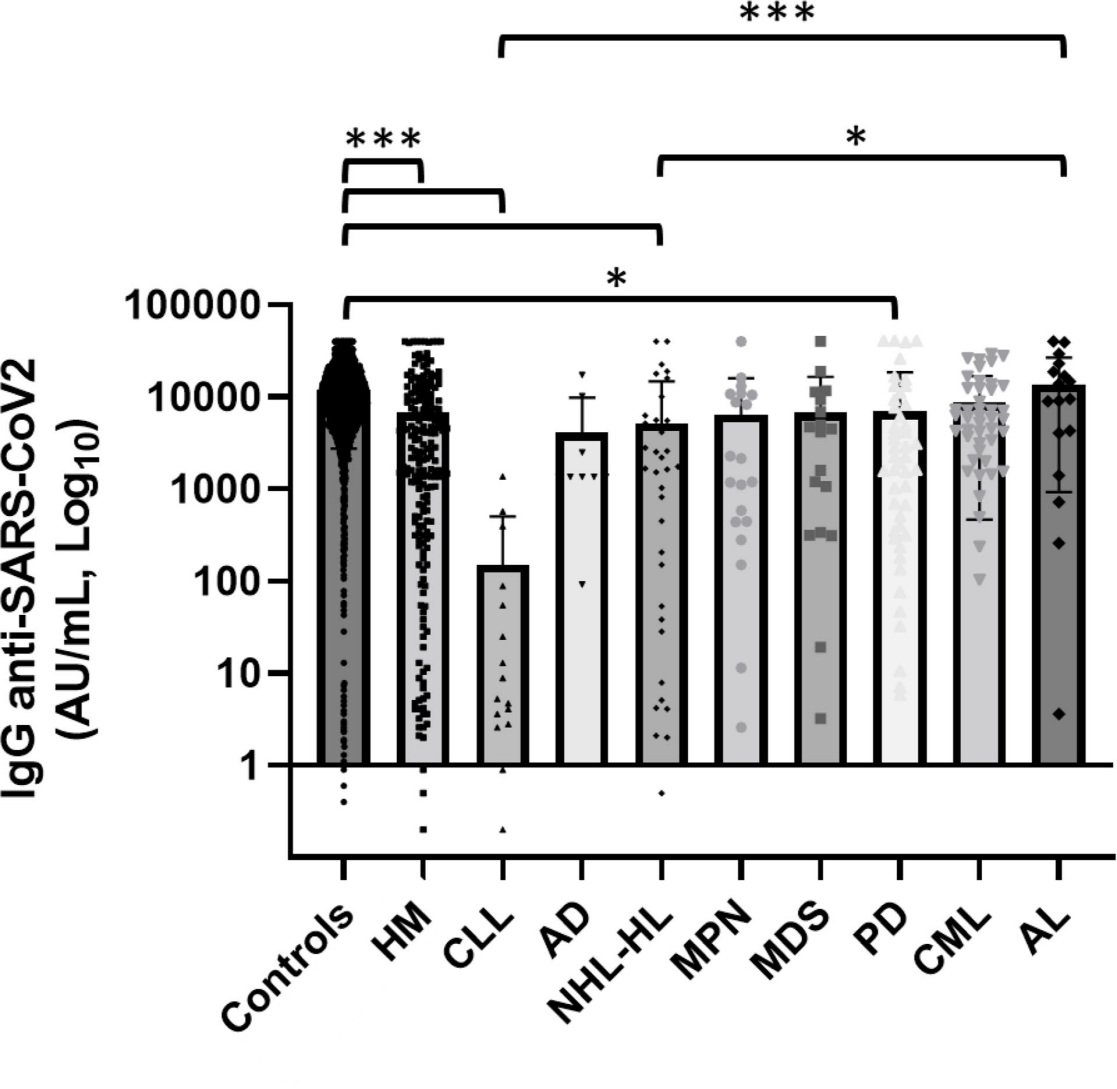
Comparison of serological response among different hematological malignancies and the comparator arm. HM, hematological malignancies; CLL, chronic lymphatic leukemia; AD, autoimmune disorder; NHL, non-Hodgkin lymphoma; HL, Hodgkin lymphoma; MPN, myeloproliferative neoplasm; MDS, myelodysplastic syndrome; PD, plasms cell disorder; CML, chronic myeloid leukemia; AL, acute leukemia; HSCT, hematopoietic stem cell transplantation. Hematological patients had a significantly lower level of anti-SARS-CoV-2 IgG than the control group (p < 0.001). CLL and lymphoma patients showed lower anti-SARS-CoV-2 IgG concentrations than AL patients (respectively p < 0.0001 and p = 0.008). Differences assessed by one-way ANOVA test (p < 0.001), Bonferroni *post-hoc* test: (*) p < 0.05; (**) p < 0.005; (***) p < 0.001. Error bars correspond to standard deviation calculated from the mean of relative concentrations. Values below black line were corresponding to anti-SARS-CoV-2 IgG lower than 10.0 AU/ml, and values of 0 AU/ml were not shown since the graph was on a log scale.

Mean concentrations of anti-SARS-CoV-2 IgG within the patients cohorts are reported in **Table 3** and **Figure 2**. We observed high variability in serological response among different hematological malignancies. Chronic lymphatic leukemia (CLL) patients had the lowest level of antibodies while acute leukemia (AL) patients had the highest seroconversion rate among the patient cohorts. The mean concentration of antibodies anti-SARS-CoV-2 observed in CLL patients was significantly lower than AL patients (p < 0.0001) and CML ones (p = 0.023). A significant difference in antibody response was noted between AL and lymphoma patients (p = 0.008). We did not observe statistically significant differences in antibody concentrations among the other malignancies. Patients affected by plasma cell disorders, myelodysplastic syndromes (MDSs), and myeloproliferative neoplasms (MPNs) showed levels of antibody concentration not different from the mean concentration of all-patient group. In these latter setting of patients, we did not investigate a statistical analysis by therapy, because of the dispersion among subgroups, but we observed a good serological response (mean concentration 12,190.7 AU/ml) among patients in maintenance therapy with lenalidomide, while patients who received ruxolitinib had a mean level of antibody concentration of 296.4 AU/ml (**Supplemental Figures 2, 3**).

**Table 3:** Concentrations of IgG anti-SARS-CoV2 in hematologic malignancies (AU/mL).

Hematologic malignancies	IgG anti-SARS-CoV2, mean	IgG anti-SARS-CoV2, median
Plasma cell disorders	7,041.3	1,595.3
Non-Hodgkin lymphoma, Hodgkin lymphoma	5,137.6	1,636.3
Chronic lymphatic leukemia	150.2	5.3
Acute leukemia	13,770	9,487
Chronic myeloid leukemia	8,700.4	6,071.8
Myelodysplastic syndrome	6,735.1	4,158
Autoimmune disorders	4,083.4	1,351.8
Myeloproliferative neoplasm	6,412	1,667.4

### 3.4 Comparison of serological response among various anticancer treatments

**Figure 3:**
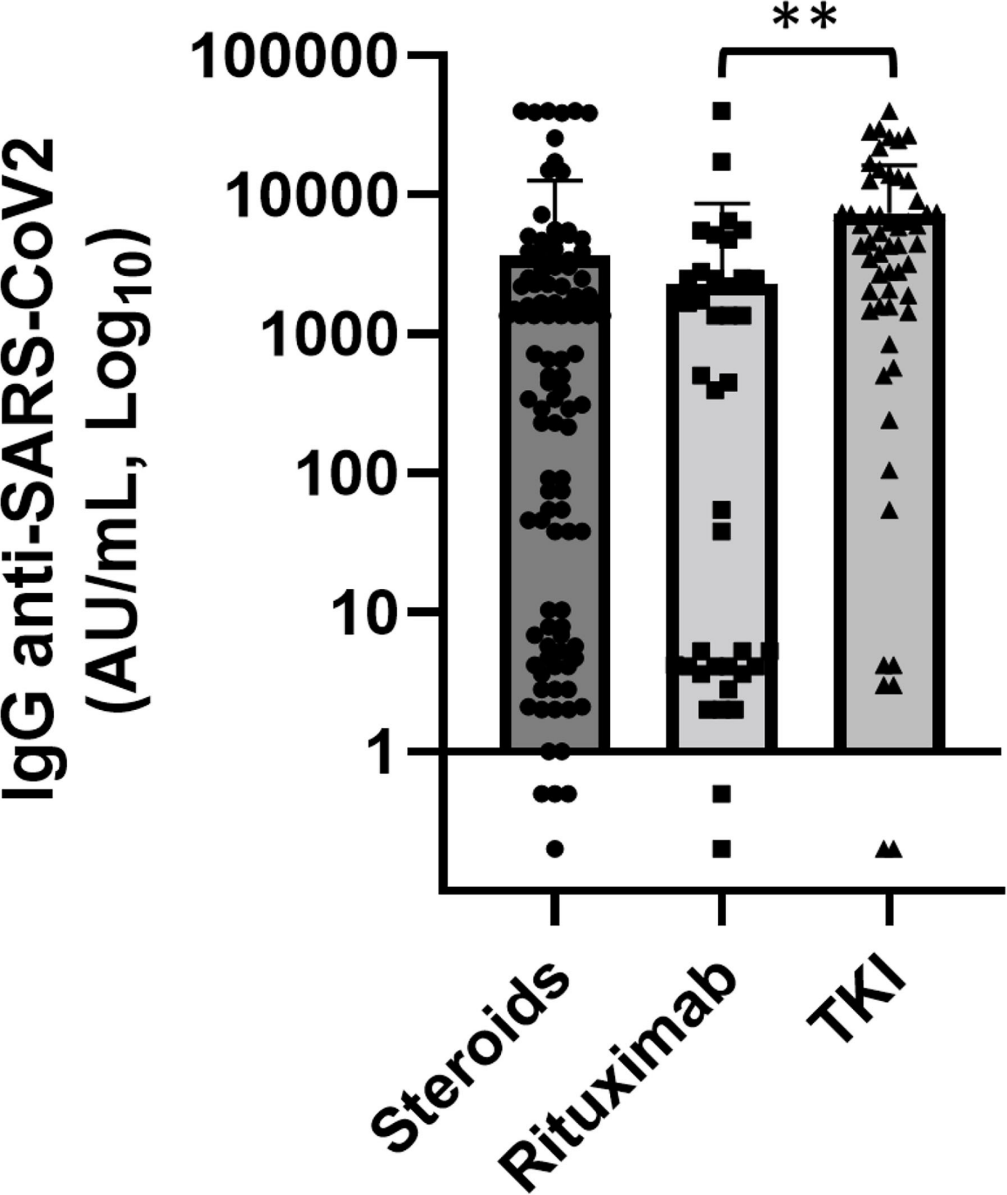
Association of anti-SARS-CoV2 IgG concentrations with various anticancer treatments. Patients treated with rituximab (n = 30) showed significantly lower levels of anti-SARS-CoV-2 IgG than patients treated with tyrosine kinase inhibitors (TKI) (n = 44) (p = 0.004). Differences assessed by one-way ANOVA test (p = 0.005), Bonferroni *post-hoc* test: (**) p < 0.005. Error bars correspond to standard deviation calculated from the mean of relative concentrations. Values below the black line correspond to anti-SARS-CoV-2 IgG lower than 10.0 AU/ml, and values of 0 AU/ml were not shown since the graph is on a log scale.

We also studied the association between treatment and levels of anti-SARS-CoV-2 IgG, especially focusing the analysis on immunosuppressive therapies, such as steroids and rituximab, and TKI for CML patients (p = 0.005 **Figure 3**).

#### 3.4.1 Steroids

**Figure 4:**
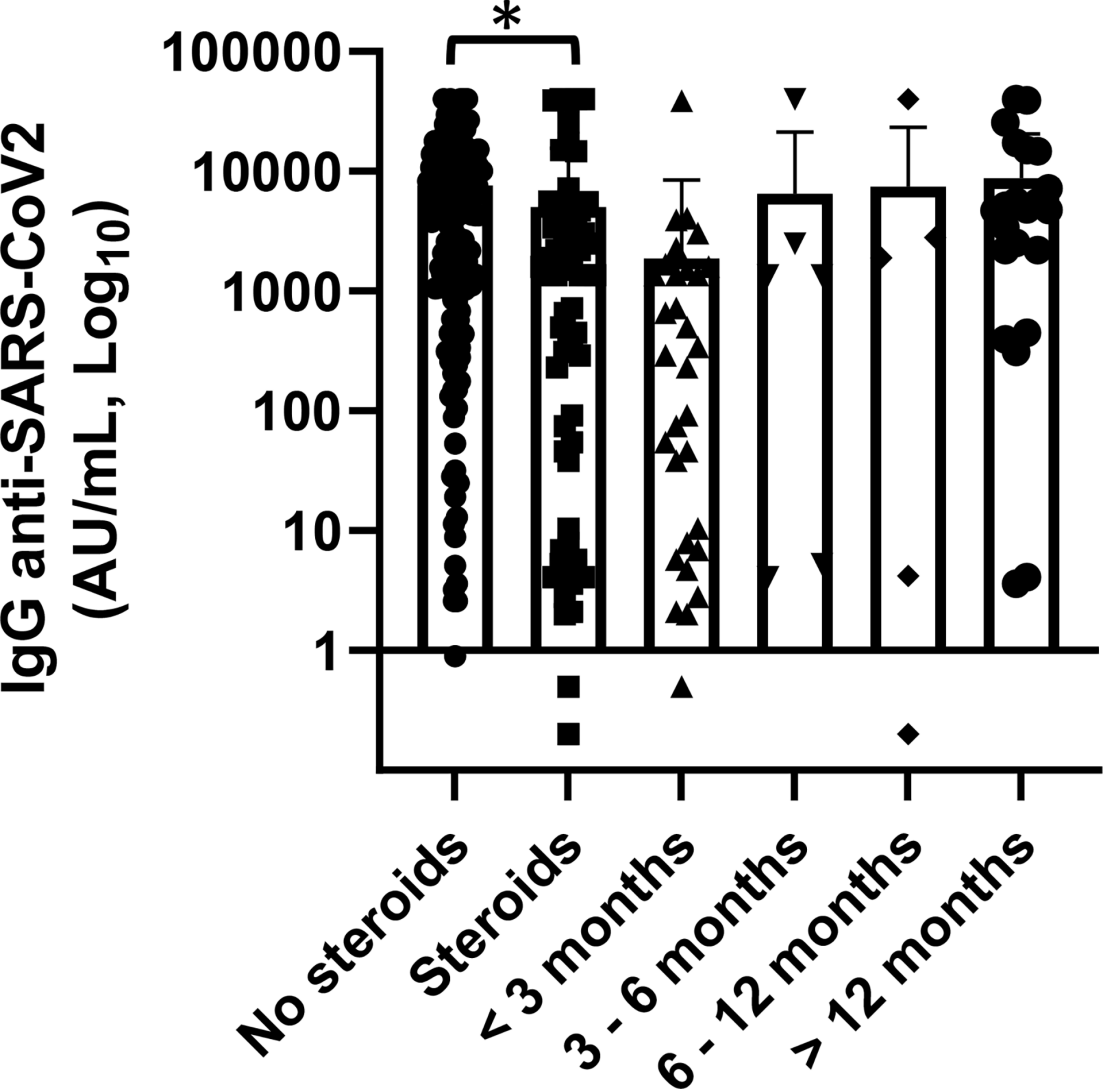
Association of anti-SARS-CoV2 IgG concentrations with steroid therapy and distance from the end of steroid treatment. Patients treated with steroid therapy (n = 70) showed significantly lower levels of anti-SARS-CoV-2 IgG than patients with did not receive them (p = 0.045). Differences assessed by t-test, (*) p < 0.05. Error bars correspond to standard deviation calculated from the mean of relative concentrations. Values below the black line correspond to anti-SARS-CoV-2 IgG lower than 10.0 AU/ml, and values of 0 AU/ml were not shown since the graph is on a log scale.

As expected, patients who had administered corticosteroids prior and at the time of vaccination had a mean concentration of anti-SARS-CoV-2 IgG significantly lower than who did not use (5,067.8 *vs*. 7,605 AU/ml, p = 0.045). Corticosteroids were part of a therapeutic program where dosage and duration of treatment were standardized according to hematological disease. No patient received only occasional dosing either reduced dosage. This setting of patients also had significantly lower serum IgG, IgA, and IgM levels (p < 0.0001, p = 0.001, p = 0.014, respectively). To note though, among 34 patients treated with corticosteroids in the last 3 months prior, vaccination 14 (41%) had a concentration of SARS-CoV-2 spike IgG below the cutoff of 50.0 AU/ml while 13 patients (38%) had a value greater than 500 AU/ml. In patients who had terminated steroid treatment for more than 3 months, the anti-SARS-CoV-2 IgG concentration was greater and it increased progressively as the distance from the end of steroid treatment increased (p = not statistically significant **Figure 4**).

#### 3.4.2 Rituximab

**Figure 5:**
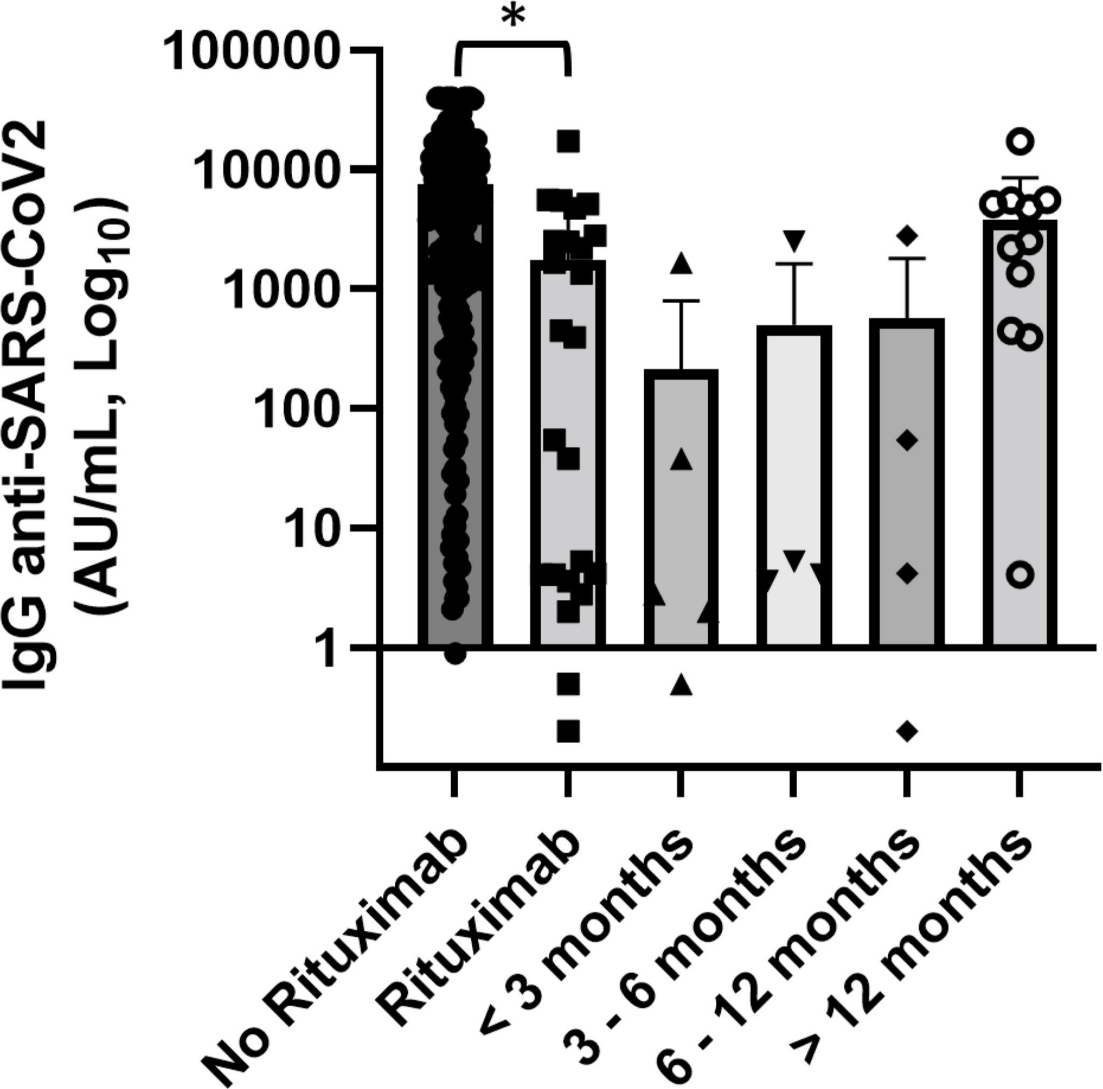
Association of anti-SARS-CoV2 IgG concentrations with anti-CD20 therapy and distance from the end of anti-CD20 treatment. Patients treated with rituximab (n = 30) showed significantly lower levels of anti-SARS-CoV-2 IgG than patients did not receive anti-CD20 treatment. Differences assessed by one-way ANOVA test (p = 0.003), Bonferroni *post-hoc* test: (*) p < 0.05. Error bars correspond to standard deviation calculated from the mean of relative concentrations. Values below the black line correspond to anti-SARS-CoV-2 IgG lower than 10.0 AU/ml, and values of 0 AU/ml were not shown since the graph is on a log scale.

Notably, antibody concentrations were very low in the setting of patients treated with rituximab compared with the rest of patients who did not use anti-CD20 (1,743 *vs*. 7,596 AU/ml) (p = 0.003). In this subgroup of patients, three affected by autoimmune diseases received rituximab as a single agent and the others 27 in combination with chemotherapy. Among 18 patients who had received rituximab in the last 12 months, only three patients had levels of antibodies greater than the cutoff, while in 12 patients who had received vaccination after more than 12 months since the last dose of rituximab the mean concentration of anti-spike IgG was 3,767.1 AU/ml and only two patients (16.6%) were seronegative (**Figure 5**). Worthy of note is that in this setting of patients the levels of total serum of IgG were significantly lower while there was not a statistically significant reduction of IgA and IgM. Thee data, in contrast with literature (15), should be confirmed by a larger case series.

#### 3.4.3 Tyrosine kinase inhibitors

We also studied the association between TKI therapy and anti-spike IgG levels in CML patients. In accordance with literature, all CML patients have demonstrated to have a good serological response (16, 17). We observed levels of antibodies greater in patients who had received second- and third-generation TKI than in patients who had used imatinib (11,301 *vs*. 6,532 AU/ml). This trend was not statistically significant; it could be influenced by different median ages between two groups of patients (66 *vs*. 57 years), and it should be confirmed on a larger sample size.

### 3.5 Association with serum immunoglobulin levels (IgG, IgA, and IgM)

We investigated the association between anti-spike IgG concentrations and total serum immunoglobulin levels (IgG, IgA, and IgM) in patients affected by plasma cell disorders and CLL, and there was no statistically significant association regarding them. Instead we noted, in lymphoma patients, a statistically significant association between anti-spike antibody levels and total serum IgG (p = 0.015) but not with IgM and IgA concentrations (Supplementary Methods, **Supplemental Figure 4**).

### 3.6 Association with hematopoietic stem cell transplantation

**Figure 6:**
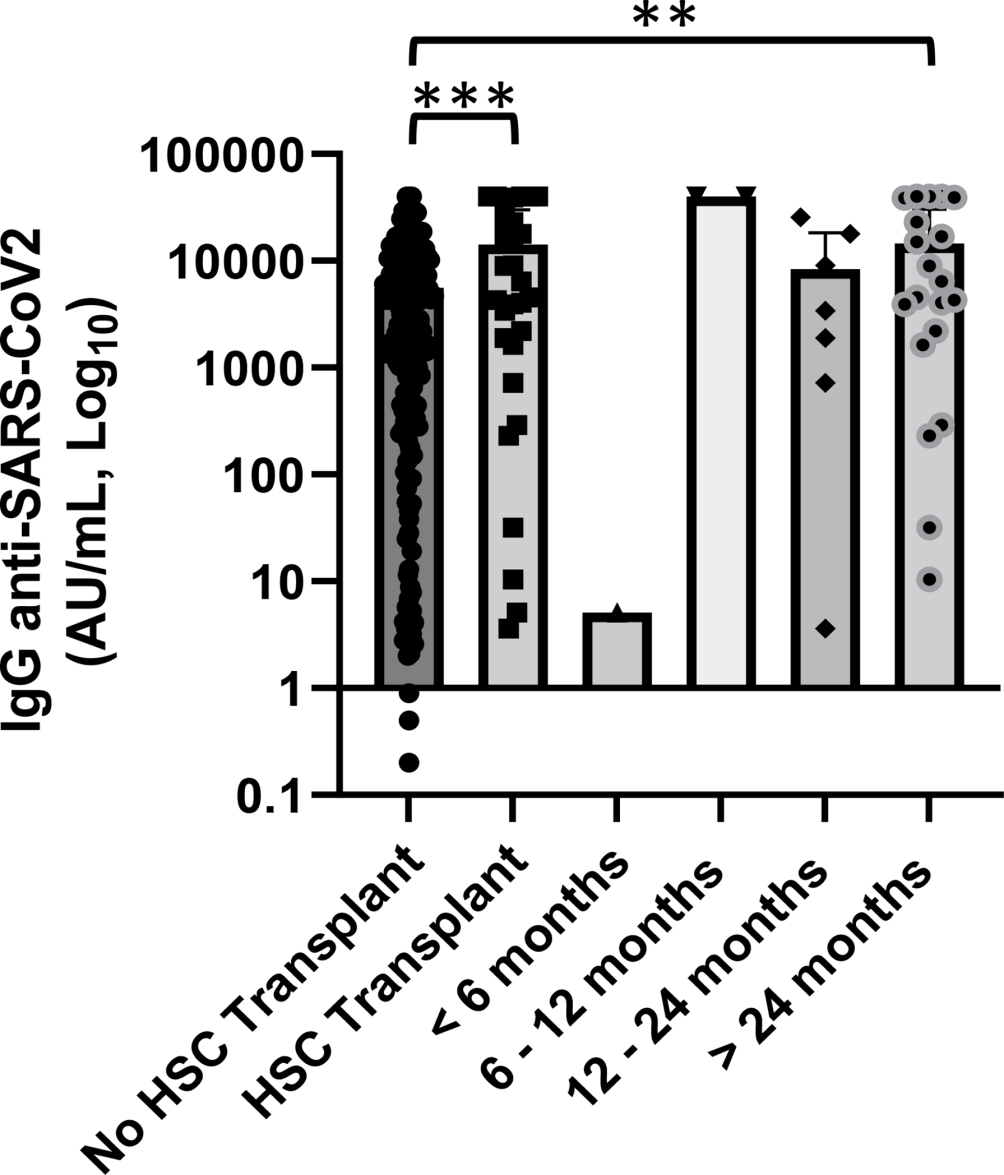
Association of anti-SARS-CoV2 IgG concentrations with hematopoietic stem cell (HSC) transplant and distance from day 0 of infusion. Patients who received an HSC transplant showed a significantly lower level of anti-SARS-CoV-2 IgG than patients who did not perform (p < 0.001). Differences assessed by one-way ANOVA test (p < 0.001), Bonferroni *post-hoc* test: (***) p < 0.001. Statistical analysis was not done for groups of patients with a median follow-up since day 0 of infusion <12 months because of small number of cases. Error bars correspond to standard deviation calculated from the mean of relative concentrations. Values below the black line correspond to anti-SARS-CoV-2 IgG lower than 10.0 AU/ml, and values of 0 AU/ml were not shown since the graph is on a log scale. **p<0.01.

Our cohort included 30 patients who performed hematopoietic stem cell transplantation, 20 autologous and 10 allogeneic, with a median follow-up since day 0 of infusion >24 months. The mean concentration of anti-SARS-CoV-2 IgG was 14,265.7 AU/ml (range 3.6–40,000 AU/ml). Among this setting of patients, only one patient had received the transplant for less than 6 months and only four of them had a concentration of anti-spike IgG below the cutoff of 50.0 AU/ml (**Figure 6**).

### 3.7 Vaccine side effects

**Figure 7:**
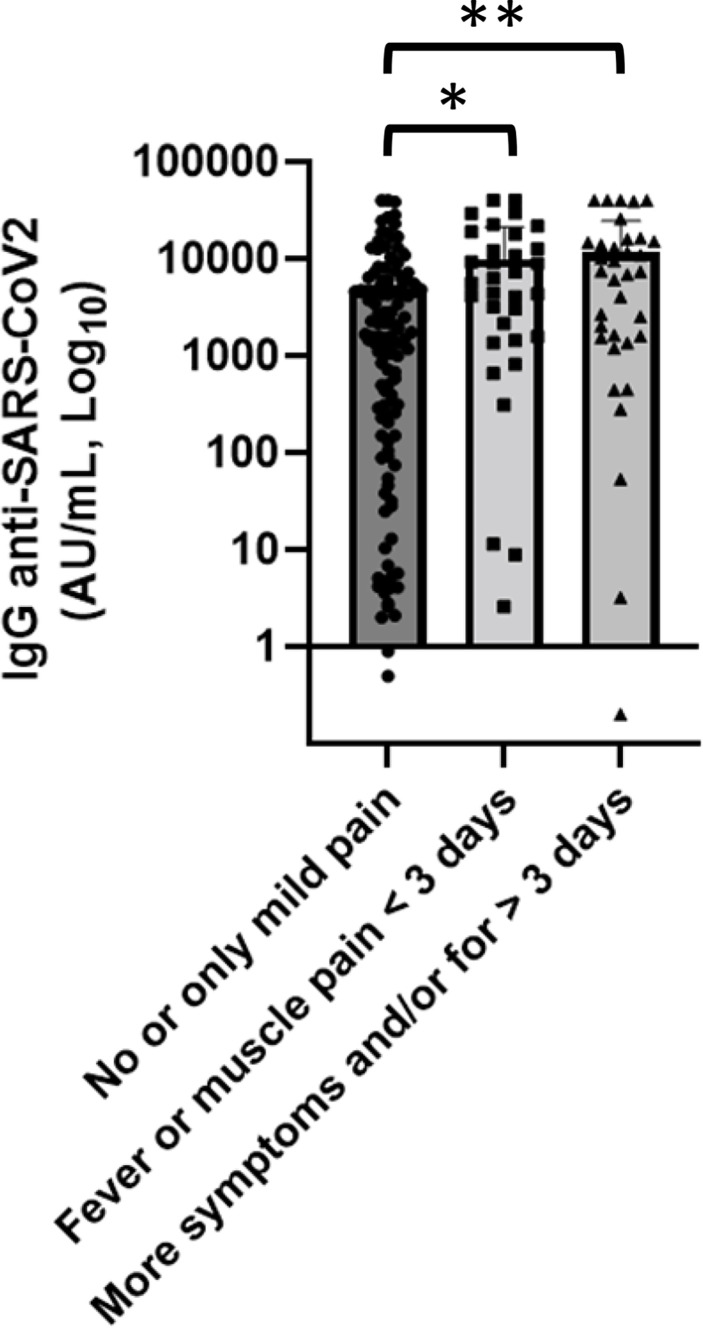
Association of anti-SARS-CoV-2 IgG concentrations with side effects. Patients who experienced more symptoms showed anti-SARS-CoV-2 IgG titer greater than patients who did not report side effect or only mild pain. Differences assessed by one-way ANOVA test (p < 0.001), Bonferroni *post-hoc* test: (*) p < 0.05; (**) p < 0.005. Error bars correspond to standard deviation calculated from the mean of relative concentrations. Values below the black line correspond to anti-SARS-CoV-2 IgG lower than 10.0 AU/ml, and values of 0 AU/ml were not shown since the graph is on a log scale.

We collected data to evaluate vaccine safety; none of the patients had experienced serious adverse event after getting the SARS-CoV-2 vaccine. A percentage of 64.8% of patients did not report any side effect or only mild pain at the injection site within a few days. Mild to moderate osteoarticular pain, fever, or asthenia occurred in 29.6% of patients. In the remaining 5.6%, lymphadenopathy or moderate thrombocytopenia was reported. We also studied the association between anti-spike IgG and side effects classified according to the number of symptoms and their duration and severity into three groups: the first group of patients, who did not report any side effect or only mild pain, had a mean concentration of anti-SARS-CoV-2 IgG of 4,817 AU/ml, patients who experienced fever or muscle pain for less than 3 days belong to the second group and had a value of anti-spike concentration of 9,943 AU/ml, while patients who reported more symptoms and/or for more than 3 days had antibody concentrations greater than the others (12,907.3 AU/ml) (**Figure 7**). In univariate analysis, patients who experienced more symptoms showed anti-SARS-CoV-2 IgG titer greater than patients who did not report side effect or only mild pain (p = 0.0001).

## 4 Discussion

Vaccination in patients with hematological malignancies is complex as the background and the characteristics of immunosuppressed states differ between patients categories. Both disease and therapy—including monoclonal antibodies—drugs with immunomodulatory effects such as corticosteroids are the main factors influencing response to vaccination and could be at a disadvantage regarding also COVID-19 vaccination. This was demonstrated for influenza, herpes zoster, hepatitis B, and pneumococcal vaccine compared with healthy controls and those with solid tumors, as well as lymphoma patients treated with anti-CD20 treatment (18, 19). Therefore, serological response to vaccine in hematological patients is considerably impaired, and this is probably not affected by different technologies and/or antigen targets utilized for the formulation of an effective vaccine but by type of disease, remission status, and treatment.

In this study, we aimed to investigate the serological response following the recommended two-dose BNT162b2 COVID-19 vaccine in a cohort of patients treated at UOC of Hematology of San Carlo Hospital in Potenza. Percentages of subjects for each hematologic disease were not in line with their expected prevalence in an unselected population, since they were enrolled in the study in a short period respecting the window of 30–45 days between the second-dose vaccine and final serological measurement according to visits scheduled by clinical practice and excluding patients who had contracted SARS-CoV-2.

Although both patients and healthy health workers performed periodical nasopharyngeal swab to investigate the presence of SARS-CoV-2 virus and who had a diagnosis of a previous exposure were not included in the study, in the absence of baseline serological data there could be an underestimation of natural infection not diagnosed of two target populations. This would not allow to exclude a partial seroconversion prior to vaccination.

Nevertheless, our data were in line with other studies that had reported a lower anti-SARS-CoV-2 serological response in hematological patients (20, 21).

Indeed, patients with hematological malignancies, compared with the control arm, had both a mean concentration of anti-SARS-CoV-2 IgG significantly lower and a seroconversion rate numerically lower, defined as percentage of patients who had had a titer of SARS-CoV-2 spike IgG below the cutoff of 50.0 AU/ml.

In terms of disease subgroups, all CLL patients showed levels of antibody titer below the mean concentration, also in only clinical surveillance patients (22).

Notably, a significant difference in serological response seen when comparing CLL patients and the overall group of patients confirmed the state of immunosuppression of these patients, also highlighted by lower levels of circulating immunoglobulin, often below the minimum value (63.6% for IgA and IgG, 75% for IgM).

Comparing serological response in hematological malignancies, only AL patients had the highest seroconversion rate among the patient cohorts and a mean antibody concentration greater than the control arm. All these patients were in remission of disease after cancer therapy. These data might be biologically plausible by a good immunological recovery after AL therapy if complete and lasting remission is achieved.

We evaluated the association between serological response and treatment, especially focusing the analysis on immunosuppressive therapies, steroids, and anti-CD20 therapy. Patients treated with steroids and rituximab showed a lower level of anti-SARS-CoV-2 spike IgG (23), and we observed that patients who received steroids had an earlier recovery than patients treated with anti-CD20 therapy.

These data, as expected, raise the question whether it is appropriate to vaccinate patients treated with immunosuppressive therapies. Our results highlight the continued need to vaccinate patients treated with steroids both since only a limited group of this setting of patients had an anti-SARS-CoV-2 spike IgG titer below the cutoff of 50 AU/ml and since also cell-mediated immune response perhaps less decisive in acting as a barrier to virus entry, but probably important for containing the progression of the infection, should be monitored.

On the other hand, patients who had received anti-CD20 treatment showed a greater and lasting decrease of humoral response induced by immunosuppressive activity especially patients treated within 12 months prior to the vaccination (24). However, normal serum IgA and IgM detected levels could indicate a selective effect of rituximab on IgG, with relative sparing of other subtypes of antibodies. Even more than patients treated with steroids in this setting of patients, it should be necessary to study both humoral and T-cell-mediated immunity to better understand the role of anti-SARS-CoV-2 vaccination in patients treated with rituximab.

Differences in anti-spike IgG levels observed among CML patients stratified according to TKI therapy (imatinib *vs*. second- and third-generation TKI) could have interesting implications on the evaluation of the effects of these drugs on the immune system, but having not reached statistical significance at the moment, they should be confirmed on a larger number of patients considering also their age.

The cohort of patients who received a stem cell transplant was very heterogeneous because it included different hematological malignancies and different types of transplant; however, a mean concentration of anti-SARS-CoV2 IgG of 14,265.7 AU/ml (greater than control arm) indicates a good immunological reconstitution after transplant. Indeed, among patients who performed a transplant for over 6 months only one had a spike IgG titer below the cutoff of 50 AU/ml. A larger number of patients would allow to evaluate the differences between auto- and allogeneic transplant and among different underlying diseases.

## 5 Conclusions

Our data, despite limitations due to the cohort rather small to properly compare multiple combinations of different malignancies and treatment, confirm what has already been reported in literature on reduced serological response in hematological patients after anti-SARS-CoV-2 vaccination. However, we found a great diversity of SARS-CoV-2 antibody response according to types of pathologies and therapies.

Our results should not lead to non-vaccination of immunosuppressed patients, also because cell-mediated immune response studies in this setting of patients are ongoing, but they should be a warning to carry out the administration of a booster dose with a different cadency according to the type of hematological malignancies and continue to adopt protective measures such as masking and social distancing to limit contagion. We think vaccinations during pandemic should be accessible to everyone as soon as possible; perhaps, repeating booster doses for immunocompromised patients, while during endemic vaccinations, could be scheduled when the best immune response is expected.

Further studies could evaluate the combined actions of both humoral and cellular immune systems, because the serological response can also, indirectly, represent T helper function, given their contribution to recruitment and activation of antibody-producing B cells, beyond their role in cellular immunity.

## Data availability statement

The raw data supporting the conclusions of this article will be made available by the authors, without undue reservation.

## Ethics statement

The studies involving human participants were reviewed and approved by Regional Ethics Committee for Basilicata (approval n° 42/2021). The patients/participants provided their written informed consent to participate in this study.

## Author contributions

SP, RN, MPi, GMa, and CC implemented the research and design of the study. They were responsible for the coordination, conduction of the study, statistical analysis, and writing of the paper. TP, GB, CMu performed the humoral vaccine response laboratory analyses. RM, LF, EL, MM, and PS performed the blood sample. All other authors were responsible for data assessment, interviewed the patients as for side effects, and collected signed informed consents and clinical data for the construction of the dataset. AB managed the control cohort. MP, GMa, and CC were responsible for the supervision of the study. All authors contributed to the article and approved the submitted version.

## Conflict of interest

The authors declare that the research was conducted in the absence of any commercial or financial relationships that could be construed as a potential conflict of interest.

## Publisher’s note

All claims expressed in this article are solely those of the authors and do not necessarily represent those of their affiliated organizations, or those of the publisher, the editors and the reviewers. Any product that may be evaluated in this article, or claim that may be made by its manufacturer, is not guaranteed or endorsed by the publisher.
