# Supplementary material for: Evaluation of serological response to anti-SARS-CoV-2 mRNA vaccination in hematological patients
Source: Front Immunol. 2022 Aug 8;13:892331. doi: 10.3389/fimmu.2022.892331 (PMC9393554; doi:10.3389/fimmu.2022.892331)
Supplement: Supplementary file 1 [file DataSheet_1.doc]

**Supplementary materials**

**Supplemental Figure 1. Association of anti-SARS-CoV-2 IgG concentrations with disease status**

*

Patients in remission of phase had significantly higher level of anti-SARS-CoV-2 IgG than patients in active disease.

Differences assessed by t-test, (*) p < 0,05. Error bars correspond to standard deviation calculated from the mean of relative concentrations. Values below black line were corresponding to anti-SARS-CoV-2 IgG lower than 10,0 AU/mL and values of 0 AU/mL were not shown since graph is on a log scale.

**Supplemental Figure 2. Association of anti-SARS-CoV-2 IgG concentrations with Ruxolitinib treatment among patients affected by Myeloproliferative Neoplasms.**

Patients treated with Ruxolitinib (N=4) had lower level of anti-SARS-CoV-2 IgG than patients did not receive (N=16), but statistical analysis was not done because of small number of cases.

**Supplemental Figure 3. Association of anti-SARS-CoV-2 IgG concentrations with Lenalidomide treatment among patients affected by Plasmacell Disorders**

Patients treated with Lenalidomide (N=12) had higher level of anti-SARS-CoV-2 IgG than patients did not receive (N=35).

Differences assessed by t-test, not statistically significant.

**Supplemental Figure 4. Association of anti-SARS-CoV-2 IgG concentrations with total serum immunoglobulin levels**

**A**

**Lymphoma**


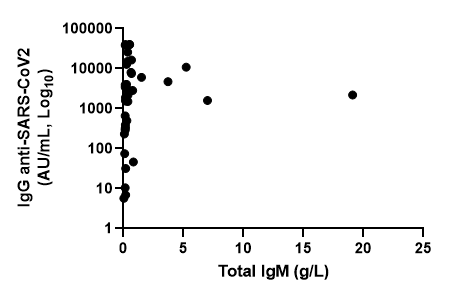


**Plasmacell disorders**

**Chronic Lymphatic Leukemia**

**B**

**C**

**
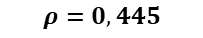
**

Association between anti-spike IgG concentrations and total serum immunoglobulin levels (IgG, IgA and IgM) in patients affected by lymphoma (A), plasmacell disorders (B) and CLL (C). Only lymphoma patients showed a statistically significant association between anti-spike antibodies levels and total serum IgG (p = 0,015). Association assessed by Pearson's correlation test.

**Supplementary Methods**

Total serum immunoglobulin levels (IgG IgA and IgM) were quantified by 800 Immage nephelometer from Beckman Instruments. The reference interval values for IgG IgA and IgM were respectively 7,51-15,6 g/L; 0,82-4,53 g/L and 7,51-15,6 g/L.
